# FSCN1 is an effective marker of poor prognosis and a potential therapeutic target in human tongue squamous cell carcinoma

**DOI:** 10.1038/s41419-019-1574-5

**Published:** 2019-05-01

**Authors:** Yue Chen, Tian Tian, Zhi-Yong Li, Chun-Yang Wang, Rong Deng, Wei-Ye Deng, An-kui Yang, Yan-Feng Chen, Hao Li

**Affiliations:** 1 State Key Laboratory of Oncology in South China, Collaborative Innovation Center for Cancer Medicine, Sun Yat-sen University Cancer Center, 510060 Guangzhou, P. R. China; 2 Department of Radiation Oncology, Sun Yat-sen University Cancer Center, 651 Dong Feng Road East, 510060 Guangzhou, Guangdong P. R. China; 3 Department of Urology, Sun Yat-sen University Cancer Center, 651 Dong Feng Road East, 510060 Guangzhou, Guangdong P. R. China; 4 Guanghua School of Stomatology, Hospital of Stomatology, Sun Yat-sen University, 510055 Guangzhou, Guangdong P. R. China; 5 Department of Radiation Oncology, University of Texas Southwestern Medical Center, 5323 Harry Hines Blvd., Dallas, TX 75390 USA; 6 Department of Head and Neck Surgery, Sun Yat-sen University Cancer Center, 651 Dong Feng Road East, 510060 Guangzhou, Guangdong P. R. China

**Keywords:** Oral cancer, Prognostic markers

## Abstract

To estimate the value of FSCN1 in evaluating the prognosis and guiding the targeted therapy for patients with tongue squamous cell carcinoma (TSCC). Using the Oncomine database, we found some genes especially FSCN1 differentially expressed between TSCC samples and tongue normal samples. So we compared FSCN1 expression between TSCC and normal cell lines and knocked down FSCN1 in TSCC cells to observe its influence on the viability and trans-migration in vitro and tumor growth in vivo. Then we measured FSCN1 expression in human cancer tissues and adjacent non-carcinoma tissues (ANT) and explored the relationship between FSCN1 expression and clinical pathological factors and prognosis in TSCC patients. We found that FSCN1 is expressed higher in TSCC cells than in normal cells. Knockdown of FSCN1 reduced TSCC cell viability and trans-migration in vitro and impaired tumor growth in vivo. FSCN1 also expressed higher in human TSCC than in ANT. In addition, FSCN1 expression was related to N classification, clinical stage and relapse. TSCC patients with over-expression of FSCN1 had worse prognosis. In conclusion, over-expression of FSCN1 indicates worse prognosis for patients with TSCC and FSCN1 may be a potential prognostic biomarker and therapeutic target in TSCC.

## Introduction

Tongue squamous cell carcinoma (TSCC) is one of the most common oral squamous cell carcinomas worldwide. TSCC is highly aggressive and characterized by early dissemination and poor prognosis^1^. The development of TSCC is a complicated and multistep process that includes a loss of cell cycle control, which may be due to genetic and epigenetic mechanisms, metabolic disturbance, dysfunction in DNA damage response and repair, and other malfunctions of the biological system^2,3^. Although some genes have been identified as related to TSCC, there are currently no reliable prognostic biomarkers or promising therapeutic targets for TSCC.

From the Oncomine database, we found that the FSCN1 gene expressed much higher in TSCC samples than in tongue normal samples. Aberrant expression of Fascin actin-bundling protein 1 (FSCN1) encodes a member of the Fascin family of actin-binding proteins. Fascin organizes F-actin into parallel bundles and is required for the formation of actin-based cellular protrusions^4^. Recent studies have shown that Fascin plays a critical role in cell migration, motility, adhesion, and cellular interactions^5,6^. The up-regulation of Fascin protein expression has been found in many cancers, such as esophageal carcinoma^7^, non-small cell lung cancer^8^, breast cancer^9^, gastric carcinoma^10^, pancreatic ductal adenocarcinoma^11^, ovarian cancer^12^, and adrenocortical carcinoma^13^, thus we hypothesized that Fascin might play a vital role in the malignant transformation of squamous cell epithelium^14^. To evaluate the relationship between the expression of Fascin protein and TSCC, we explored the expression and function of FSCN1 in TSCC cells in vitro and in vivo. We also analyzed the relationship between FSCN1 expression and the prognosis of 106 TSCC patients.

## Materials and methods

### Ethics statement

The Ethics Committee of Sun Yat-sen University Cancer Center approved use of the tumor specimens used for this research and the TSCC patients signed consent forms.

### Patients and tumor tissue samples

The pathological specimens in this research were collected from 106 patients who were diagnosed with TSCC and then underwent surgical resection at the department of head and neck surgery of Sun Yat-sen University Cancer Center between 2005 and 2009. Patients without an accurate tumor size after biopsy and with a second primary tumor at the time of surgery or before were excluded. All of the patients underwent biopsy before surgery and the Pathology Department of Sun Yat-sen University Cancer Center confirmed the diagnosis of TSCC according to the World Health Organization (WHO) histological criteria. Clinicopathological records of the patients were reviewed based on the American Joint Committee on Cancer (AJCC) Cancer Staging Manual (Eighth Edition). The median follow-up time was 110 months (range = 4–148 months). Disease-free survival time (DFS) (defined as the time from surgery to the date of relapse or metastasis) and overall survival (OS) (defined as the time from surgery to the date of death or the last follow-up) were used as measures of prognosis. Detailed information on the 106 patients is shown in Supplemental Table 1.

### Database

The Oncomine microarray database (http://www.oncomine.org) was used to screen FSCN1 expression in the paired TSCC samples. “FSCN1” was used as a keyword in the Oncomine search, “Cancer vs. Normal Analysis” was used as the primary filter, and “TSCC vs. Normal Analysis” was chosen as the analysis type. The up-regulated mRNA levels of FSCN1 were shown in multiple data sets including those from the Talbot, Estilo, Ye, and Kuriakose datasets. The FSCN1 expression data were log-transformed, median-centered per array, and the standard deviation (SD) was normalized to one per array.

### Cell lines and cell culture

The human TSCC cell lines (CAL-27, SCC-9, SCC-15, SCC-25, and Tca8113) were cultured in Dulbecco’s Modified Eagle’s Medium (DMEM; Invitrogen, Carlsbad, CA, USA) supplemented with 10% fatal bovine serum (FBS; Sigma-Aldrich, St. Louis, MO, USA). TSCC cells were maintained under a humidified 5% CO_2_ atmosphere at 37 °C. All of the cells tested negative for mycoplasma contamination and were authenticated based on STR fingerprinting before use.

### RNA isolation and qPCR analysis

Total RNA was isolated with TRIzol regent (Life Technologies, Gaithersburg, MD, USA) and then reverse transcribed with an iScript cDNA Synthesis Kit (Bio-Rad, Hercules, CA, USA). The resulting complementary DNA was analyzed by qPCR performed with SYBR reagent using the IQ5 PCR system (Bio-Rad, Hercules, CA, USA), and data were normalized to β-Actin. The sequences of the primers used were: FSCN1 (primer with non-overlapping introns: forward: 5′-ccagggtatggacctgtctg-3′, reverse: 5′-gtgtgggtacggaaggcac-3′; primer with overlapping introns: forward: 5′- ccgaccaccgcttcctg-3′, reverse: 5′-gcagacaggtccataccctg-3′.); and β-Actin (forward: 5′-ctgggacgacatggagaaaa-3′, reverse: 5′-aaggaaggctggaagagtgc-3′).

### Western blot (WB) analysis

The fresh frozen tissues were lysed in RIPA lysis buffer, and the lysates were cleared by centrifugation. The proteins were then transferred onto polyvinylidene fluoride membranes and incubated with rabbit anti-FSCN1antibody or mouse anti-β-Actin antibody at 4 °C overnight at 1/1000 dilution and 1/50,000 dilution, respectively^6^. Afterward, the membranes were incubated with secondary antibody at room temperature for 30 min and visualized using the ECL system (Santa Cruz Biotechnology, Santa Cruz, CA, USA)^15^.

### Immunoblotting and Immunohistochemical analysis

Immunoblotting and Immunohistochemical (IHC) analysis were conducted with standard procedures as previously described^16^. Blotting membranes were stripped and re-probed with anti-β-Actin antibody as a loading control. Two pathologists independently reviewed the stained sections without knowing the clinical diagnosis. For IHC evaluation, the pathologists evaluated the percentage of positive cells as well as staining intensity in five random high power fields and then took the average. FSCN1-positive signals primarily located in the cell cytoplasm, which were counted according to the brown diaminobenzidine precipitate. The staining index was determined by the proportion of positively stained tumor cells (1, <25%; 2, 25–50%; 3, 50–75%; or 4, 75–100%) multiplied by the intensity of staining (0, 1, 2, or 3). The expression levels of FSCN1 were considered high (>5) or low (≤5) based on the final scores generated by multiplying the staining proportion scores by the staining extent scores. The following antibodies were used for immunoblotting or IHC analysis: FSCN1 (1:100; #ab126772, Abcam, Cambridge, MA, USA); Ki67 (1:500, #RM-9106-S0, Thermo Fisher Scientific, Waltham, MA, USA); β-Actin (1:1000, #3700, Cell Signaling Technology, Danvers, MA, USA).

### RNAi assay and lentiviral transduction

Short hairpin RNA (shRNA) directed against FSCN1 was ligated into the pLV12 vector (GenePharma, Shanghai, China). Lentiviruses were generated by transfecting lentiviral vector pLV12 together with the packaging vector psPAX2 and envelope plasmid pMD2.G into TSCC cells, and the infected cells (CAL-27 or SCC-25 cells) were selected with 3 μg/ml puromycin over 2 weeks. The sequences targeting FSCN1 were: 5′- ggtcaacatctacagcgtcac -3′(#1) and 5′- gcgcctacaacatcaaagact -3′(#2). The scramble shRNA was used as the negative shRNA control.

### Cell viability and colony formation assays

CAL-27 and SCC-25 cells were implanted in 96-well plates at a density of 4,000 cells per well after transfection. Cellular viability was measured using an MTT assay at 1, 2, 3, 4, 5, and 6 days and the absorbance values were measured at 490 nm with a spectrophotometric plate reader. For the colony formation assay, each cell line was plated in a 6-well plate at a density of 500 cells/plate and cultured for 14 days in a humidified 5% CO_2_ incubator at 37 °C. The colonies were fixed with methanol and stained with 0.5% crystal violet solution as described previously^17^. Then, the colonies were photographed and counted. All of the assays were done in triplicate and repeated three times.

### Trans-migration assay

CAL-27 and SCC-25 exponentially growing cells were trypsinized and counted, and then implanted in the top chamber (BD Biosciences, San Jose, CA, USA) at a density of 2 × 10^4^ cells per well in medium supplemented with 1% FBS. Medium supplemented with serum was used as a chemical attractant in the lower chamber. The cells were incubated for 20 h and cells that did not tans-migrate through the pores were removed with a cotton swab. Cells on the lower surface of the membrane were stained and counted in three random fields. The trans-migration assay was done in triplicate and repeated three times.

### Cancer xenograft model and tumorigenicity assay

The entire animal experiments were performed in accordance with a protocol approved by our institutional Animal Care and Use Committee. Female BABL/c nude mice (4–5 weeks old) were obtained from the Animal Center of Guangdong Province (Guangzhou, China). For the in vivo tumorigenesis study, FSCN1-SC or shFSCN1 cells (2 × 10^6^, in PBS) were subcutaneously injected into the flanks of nude mice (five mice/group). Tumor size was measured every four days using a caliper, and tumor volume was calculated using the standard formula V = length × width2/2. Mice were killed when they met the institutional euthanasia criteria for tumor size and overall health condition on the 32nd day. The tumors were removed, photographed, and weighed. Sections were stained with hematoxylin and eosin (H&E) according to standard procedures.

### Statistical analysis

A paired-samples *t* test was used to compare FSCN1 mRNA and protein levels in cancer tissues and the matched adjacent non-carcinoma tissues, and to compare viability and trans-migration in FSCN-SC and shFSCN1 cells in vitro and in vivo. The Chi-square test and Mann–Whitney test were used to evaluate the relationship between FSCN1 expression and clinicopathological features. Kaplan–Meier curves and the log-rank test were used to determine disease-free survival and overall survival analysis. Cox regression analysis was performed to determine hazard ratios. A two-sided *p* value of <0.05 was considered statistically significant. All of the data analysis was performed with SPSS 24.0 software (SPSS Inc., Chicago, IL, USA)

## Results

### Expression of FSCN1 in TSCC from Oncomine database

**Fig. 1:**
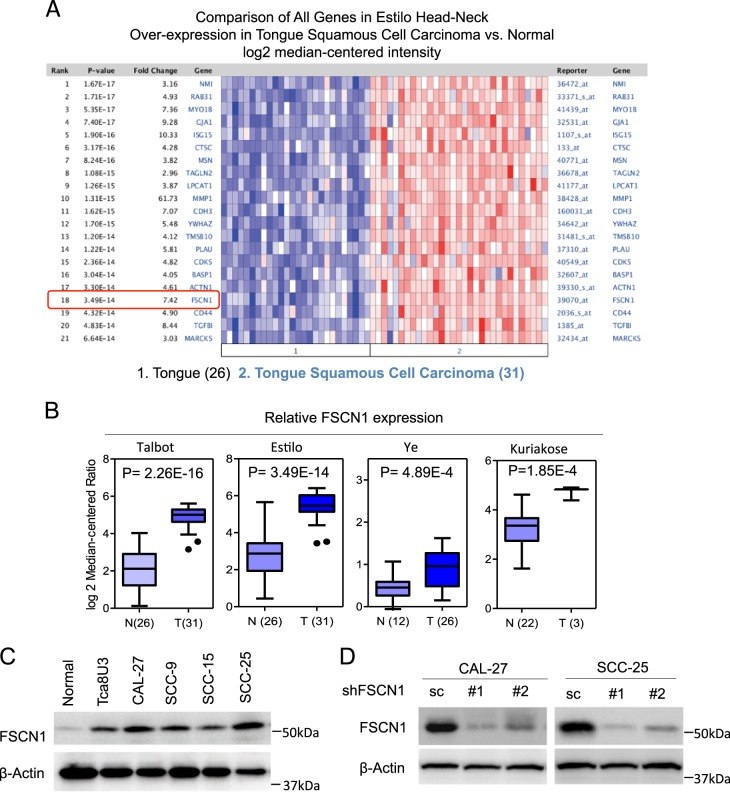
The expression of Fascin actin-bundling protein 1 (FSCN1) in tongue squamous cell carcinoma (TSCC) **a** Comparison of all of the genes over-expressed in tongue squamous cell carcinoma vs. normal tissues in the *Estilo* study. **b** Relative FSCN1 expression in tongue squamous cell carcinoma vs. normal tissues in the *Talbot*, *Estilo*, *Ye*, and *Kuriakose* databases. FSCN1 is overexpressed in human TSCC tissues (T) compared to the adjacent normal tissues (N) in TSCC microarray data sets available from Oncomine. **c** Immunoblotting analysis of FSCN1 protein levels in five TSCC cell lines and normal tongue tissue. **d** Immunoblotting evaluates the knockdown efficiency of FSCN1 with two unique shRNAs (#1, #2) in CAL-27 and SCC-25 cells. Normal: normal tongue tissue; Scramble (sc): the lentiviral vector with a scrambled sequence that does not target any mRNA. β-Actin was included as a loading control. All statistical analyses were performed using Student paired *t* test. All statistical tests were two-sided. Data is presented as mean ± S.D. **p < 0.01 vs. the corresponding control groups

Using the Oncomine database, we found a study by Estilo et al. indicated that the FSCN1 gene expressed 7.42-times higher in TSCC samples (31 samples) than in tongue normal samples (26 samples) (Fig. 1a). We also found the results from studies by Talbot, Ye, and Kuriakose consistent with Estilo (Fig. 1b). These studies all agreed that FSCN1 expression was higher in TSCC samples than in tongue normal samples (*p* < 0.05).

### FSCN1 expression in TSCC cell lines

TSCC cell lines, including Tca8U3, CAL-27, SCC-9, SCC-15, and SCC-25, were cultured under optimal conditions. Total protein was extracted from the TSCC cell lines to compare FSCN1 protein in normal tissues and TSCC cell lines via Western blotting assay. Results showed that the FSCN1 protein was up-regulated in TSCC cell lines, particularly in the CAL-27 and SCC-25 cell lines (Fig. 1c). Because FSCN1 was an over-expressed gene, it was more representative to select the cell lines with high expression level for the next step experiment, so we chose CAL-27 and SCC-25 cell lines for the following in vivo and in vitro experiments.

### Effects of FSCN1 knockdown on viability and trans-migration in vitro

**Fig. 2:**
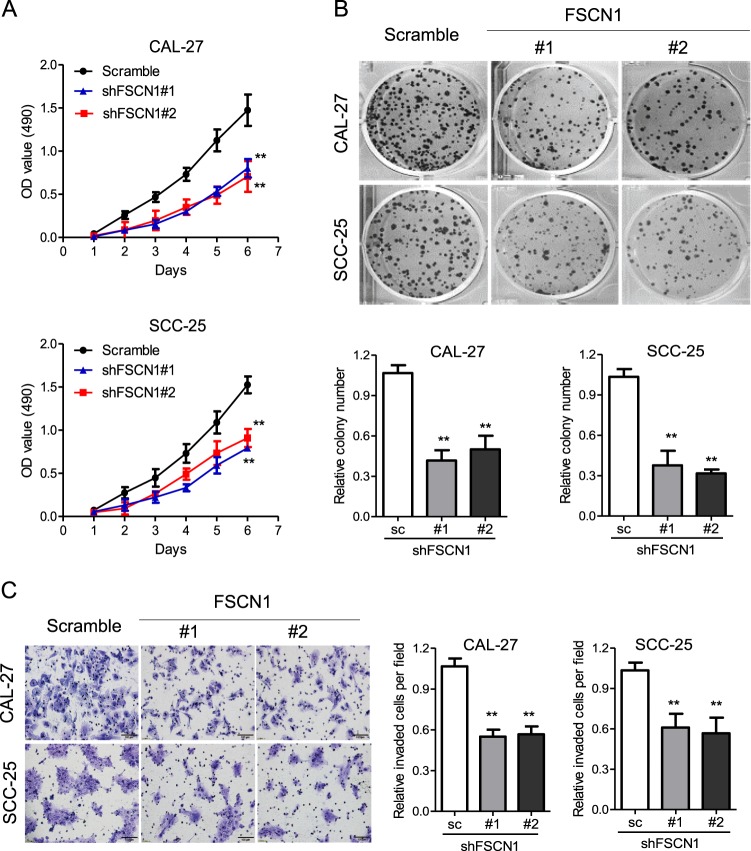
Knockdown of FSCN1 inhibited cell viability and trans-migration in CAL-27 and SCC-25 cells. **a** The cell viability of the indicated cells knocked down FSCN1 was determined by MTT assay. **b** Images (upper panel) and quantification (lower panel) of the indicated cells treated with melatonin (14 days) were analyzed in a clonogenic assay. **c** Images (left panel) and quantification (right panel) of the indicated cells treated with melatonin for 24 h were analyzed in a transwell matrix penetration assay (Scale bars, 100 μm). Scramble (sc): the lentiviral vector with a scrambled sequence that does not target any mRNA. shFSCN #1 and #2: FSCN1 with two unique shRNAs (#1, #2) in CAL-27 or SCC-25 cells. All statistical analyses were performed using Student paired *t-*test. All statistical tests were two-sided. Data is presented as mean ± S.D. **p < 0.01 vs. the corresponding control groups

To clarify the relationship between FSCN1 expression and the viability and trans-migration of TSCC cells, we knocked down FSCN1 by infecting lentiviruses to ensure that shFSCN1 had lower FSCN1 expression (Fig. 1d). Then, we examined the viability of the negative shRNA control cells (sc) and FSCN1-knockdown cells (#1 and #2) in CAL-27 and SCC-25 cell lines. MTT and colony formation assays revealed that the viability of FSCN1-knockdown cells was significantly inhibited (Fig. 2a, b). It is known that regional lymph node metastasis is an adverse prognostic factor of TSCC. The ability of cancer cells to trans-migrate from the primary site into distant tissues is a crucial step in the formation of metastasis. Therefore, we tested the ability to trans-migrate of FSCN1-knockdown cells using a transwell migration assay. The number of cells trans-migrating through the membranes was significantly reduced in FSCN1-knockdown cells in comparison to the control cells, suggesting a pro-metastasis role for FSCN1 (Fig. 2c).

### Effect of FSCN1 knockdown on proliferation in vivo

**Fig. 3:**
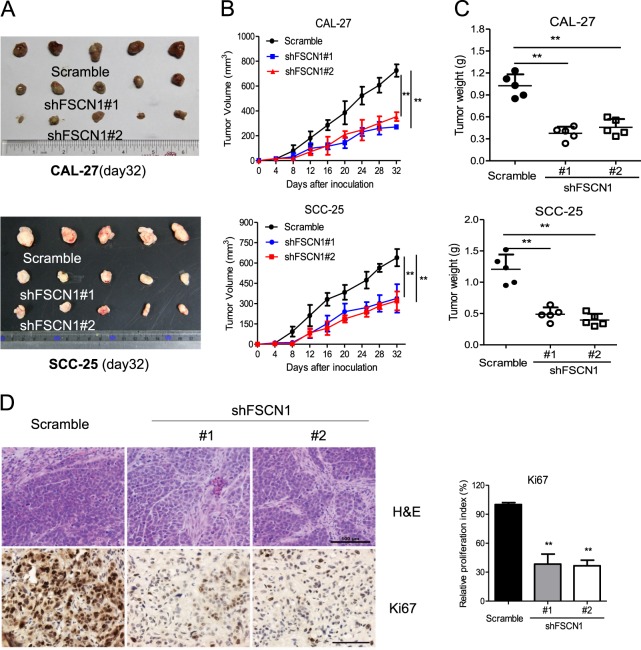
Knockdown of FSCN1 impaired tumor growth in a xenograft mouse model. **a** Photograph and comparison of excised tumor size in CAL-27 or SCC-25 cell lines. **b** The nude mice were injected subcutaneously with CAL-27 or SCC-25 cell lines. The tumor sizes were measured throughout the experiment to evaluate FSCN1 knockdown effects. **c** Weights of tumor tissues removed from mice injected shFSCN1 TSCC cells or control TSCC cells on day 32. **d** Paraffin- embedded tumor sections were stained with H&E or anti-Ki67 antibody (Scale bars, 100 μm). Quantification of proliferation index of Ki-67 in TSCC tumors was shown. Scramble (sc): the lentiviral vector with a scrambled sequence that does not target any mRNA. shFSCN #1 and #2: FSCN1 with two unique shRNAs (#1, #2) in CAL-27 or SCC-25 cells. All statistical analyses were performed using Student paired *t*-test. All statistical tests were two-sided. Data is presented as mean ± S.D. **p < 0.01 vs. the corresponding control groups

To clarify the relationship between FSCN1 expression and its influence on tumorigenic capacity in nude mice, we injected FSCN1-knockdown and control cells into mice and marked the tumor volume over time. On the 32nd day, we observed that the tumor proliferation of nude mice injected with FSCN1-knockdown cells was slower than the control cells both in CAL-27 and SCC-25 cell lines (Fig. 3a). In addition, consistent results from the tumor volume-time curves and the tumor weight scatter plot were obtained (Fig. 3b, c). When the tumors were cut into slices and stained by immunohistochemistry, we found that the tumor cells were less dense and exhibited lower proliferation in the control cells than in the FSCN1-knockdown cells (Fig. 3d, e).

### Expression of FSCN1 mRNA and protein in human TSCC tissues

**Fig. 4:**
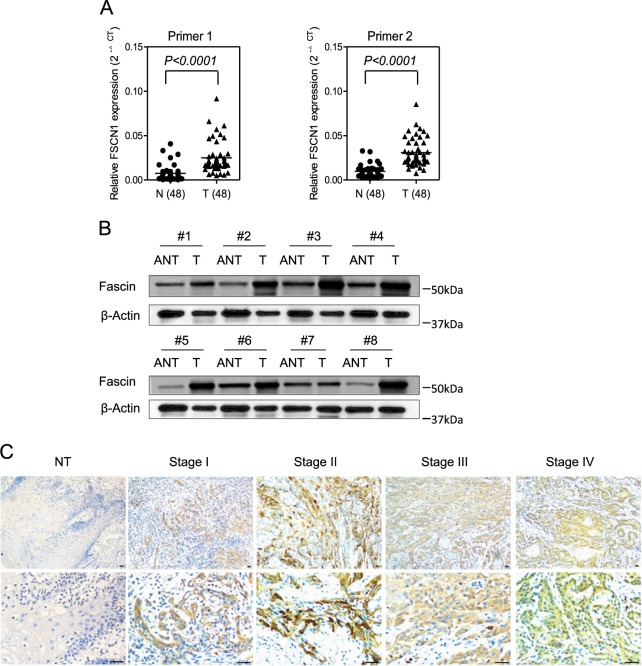
The expression of FSCN1 in human tongue squamous cell carcinoma (TSCC). **a** Expression of FSCN1 mRNA in paired TSCC tissues (*N* = 48) was measured by qRT-PCR assay with primers with overlapping introns (Primer 2) or non-overlapping introns (Primer 1). The relative FSCN1 mRNA expression levels significantly increased in TSCC tissues (T) compared with those of the matched adjacent normal tissues (N) (*p* < 0.0001). **b** Expression of Fascin in paired TSCC tissueswas measured by western blotting (*N* = 8). The relative FSCN1 protein expression levels significantly increased in TSCC tissues (T) compared with those of the matched adjacent non-carcinoma tissues (ANT) (*p* < 0.0001). β-Actin was measured as the loading control. **c** Representative immunohistochemical staining showing positive staining in TSCC tissues with different stages and negative staining in normal tongue tissue(NT) (Scale bars, 100 μm). All statistical analyses were performed using Student paired *t-*test. All statistical tests were two-sided

To analyze FSCN1 mRNA and protein expression in TSCC tissues (T) and the adjacent non-carcinoma tissues (ANT), we performed qRT-PCR assay and found that the expression of FSCN1 mRNA was demonstrably higher in T than in ANT in 48 pairs of fresh frozen tissues from TSCC patients (*p* < 0.0001) (Fig. 4a). Consistent results were obtained when testing FSCN1 protein (Fascin) expression via western blotting in eight pairs of fresh frozen tissues from TSCC patients (Fig. 4b).

### Immunohistochemical analysis of FSCN1 expression in TSCC samples and the relationship to clinicopathological parameters

To further investigate FSCN1 expression in TSCC, we performed IHC analysis of 106 paraffin-embedded TSCC tissues, involving 95 tissues with both carcinoma tissues and the matched ANT tissues. Results showed that all of the tumor tissues expressed significantly higher Fascin protein than the matched ANT tissues. Representative IHC-stained slides displaying the adverse connections between tumor clinical state and FSCN1 expression are shown in Fig. 4c. Then, we analyzed the potential correlation between FSCN1 protein expression in 106 TSCC surgical specimens and the clinicopathological features of the TSCC patients. Table 1 shows that 62.2% (69/106) of cases exhibited high FSCN1 expression, while 34.9% (37/106) of cases showed relatively low FSCN1 expression. The samples consisted of 34.9% (37/106) cases of clinical stage I, 29.2% (31/106) cases of clinical stage II, 19.8% (21/106) cases of clinical stage III, and 16.0% (17/106) cases of clinical IV.

**Table 1:** Correlation between FSCN1 expression and clinicopathological variables of 106 TSCC cases

Clinicopathological variables	Number of each group	FSCN1 expression		*p* value
		Low	High	
Age (years)				0.788
≤45	27	10	17	
>45	79	27	52	
Gender				0.497
Male	62	20	42	
Female	44	17	27	
Differentiation state				0.376
well	72	27	45	
Morderate	28	6	22	
Poor	6	3	3	
T classification				0.611
T1 + T2	83	30	53	
T3 + T4	23	7	16	
N classification				0.016
N0	80	33	47	
N1 + N2 + N3	26	4	22	
M classification				0.349
M0	105	36	69	
M1	1	0	1	
Clinical stage				0.047
I	37	14	23	
II	31	14	17	
III	21	8	13	
IV	17	1	16	
Relapse				0.003
Yes	30	4	26	
No	76	33	43	
Death				0.064
Yes	32	7	25	
No	74	30	44	

The relationship between the clinical and pathological characteristics of TSCC patients and the level of FSCN1 expression are described in Table 1. Results showed that the expression level of FSCN1 was significantly correlated with multiple variables, including N classification (*p* = 0.016), clinical stage (*p* = 0.047) and relapse (*p* = 0.003), but not with age (*p* = 0.788), gender (*p* = 0.497), differentiation state (*p* = 0.376), T classification (*p* = 0.611), M classification (*p* = 0.349), or death (*p* = 0.064).

### FSCN1 expression and patient survival analysis

**Fig. 5:**
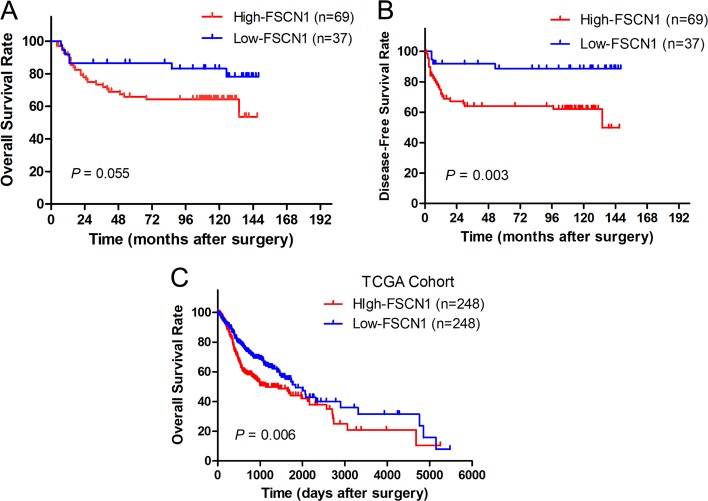
Kaplan–Meier survival curves for TSCC patients with high FSCN1 expression (red line) vs. low FSCN1 expression (blue line). **a** Relationship between FSCN1 expression and OS of TSCC patients in our own cohort. **b** Relationship between FSCN1 expression and DFS of TSCC patients in our own cohort. **c** Relationship between FSCN1 expression and OS of head and neck carcinoma patients in TCGA cohort. Cumulative survival rates were estimated by the Kaplan–Meier method and compared by the log-rank tests. All statistical tests were two-sided

The potential prognoses of FSCN1 for DFS and OS in TSCC patients was evaluated by comparing the survival time and disease-free survival time of patients with high FSCN1 expression to patients with low FSCN1 expression. The data identified from TCGA database demonstrated that high expression of FSCN1 suggested poor OS (*p* *=* 0.006) in 496 TSCC patients (Fig. 5c). As for the 106 TSCC patients in our hospital, higher FSCN1 expression experienced a trend towards lower OS (*p* *=* 0.055), and had decreased disease-free survival time (*p* *=* 0.003), as shown in Fig. 5a, b.

To investigate the independent prognostic value of FSCN1 expression levels, univariate and multivariate Cox regression analyses were performed (Table 2). Results revealed that FSCN1 expression and clinical state were associated with disease-free survival of TSCC patients. Further, multivariate Cox regression analysis showed that FSCN1 expression and clinical state were correlated with poor DFS in TSCC patients.

**Table 2:** Univariable and multivariable analysis of disease-free survival of TSCC patients

Variables	Univariate analysis			Multivariate analysis		
	HR	95%CI	*p* value	HR	95%CI	*p* value
FSCN1 expression (low vs. high)	4.345	1.506–12.539	0.007	3.893	1.333–11.370	0.013
Age (≤45 vs. >45)	1.026	0.456–2.305	0.951			
Gender (male vs. female)	0.746	0.364–1.528	0.423			
Differentiation stage	1.149	0.628–2.100	0.652			
Clinical stage (I + II vs. III + IV)	2.009	0.967–4.171	0.061			
N classification (N0 vs. N1 + 2 + 3)	2.900	1.360–6.185	0.006	1.417	1.030–1.948	0.032

## Discussion

TSCC is highly aggressive characterizing by early dissemination and poor prognosis^1,18^ with no good therapeutic effect at present. We know that valid prognostic biomarkers can improve treatment planning and forecast outcomes of patients with TSCC. Moreover, effective biomarkers can increase knowledge of the malignant biological behaviors of tumors so that patient outcomes can be predicted earlier and more precisely. However, at present there are no enough studies of marker and treatment targets on the prognosis of TSCC. It is known that Oncomine is a powerful cancer microarray database and consolidated data mining platform that can aid in the identification of new biomarkers or therapeutic targets. We first mined the differentially expressed genes between TSCC and normal tongue tissues in the Oncomine database. Based on the results of Estilo et al., we identified a differentially expressed gene between TSCC and normal tongue tissues. The FSCN1 gene was expressed much higher in TSCC tissues than in normal tongue tissues. As the up-regulation of Fascin protein expression has been confirmed in many cancers, such as esophagus carcinoma^7^, non-small cell lung cancer^8^, breast cancer^9^, gastric carcinoma^10^, pancreatic ductal adenocarcinoma^11^, ovarian cancer^12^, and adrenocortical carcinoma^13^, we hypothesized that FSCN1 might be a biomarker in TSCC. We then verify our hypothesis by studying TSCC cell viability and trans-migration in vitro, tumor growth in vivo and FSCN1 expression and patient survival analysis.

Firstly, we detected FSCN1 expressed higher in all the five different TSCC cell lines than tongue normal tissues. In order to observe more obvious changes of FSCN1 expression after knocking down, we selected the CAL-27 and SCC-25 cell lines for the following experiment as for their higher expression of FSCN1. With in vitro and in vivo experiments, we found that FSCN1 might be an important gene in promoting tumor growth and metastasis. The findings of Park et al. in ovarian cancer cells, Kim et al. in gastric cancer cells, and Alam et al. and Lee et al. in oral cancer cells were in accordance with our results^10,19–21^. Alternatively, results from Rodrigues et al. showed that the knockdown of Fascin did not impair the viability or proliferation of oral cancer cells^6^. These contradictory results suggest that there might be tumor heterogeneity in different primary sites or diverse types of oral epithelia. This greatly reinforces our research strategy of solely focusing on TSCC to reduce confounding factors and achieve more reliable conclusions.

Then, we detected FSCN1 expression at the levels of transcription and translation in a relatively large number of human TSCC tissue specimens and matched ANT specimens. In previous studies, researchers have analyzed FSCN1 expression in cancers, such as esophageal, gastric, and oral carcinoma^7,21,22^. These studies also found differential expression between carcinoma tissues and normal tissues using PCR and Western blotting methods. In the immunohistochemical analyses, FSCN1 expression was increased in all of the TSCC tissues (95/95) in comparison with the matched adjacent non-carcinoma tissues. Our experimental results confirmed the data obtained from the Oncomine database. Shimamura et al. studied Fascin expression in oral dysplasia and carcinoma in situ via immunohistochemical analysis and reported that Fascin was significantly higher in dysplasia and malignant disease than in benign disease^23^. Their result strengthens our finding that FSCN1 may improve the diagnostic accuracy of oral dysplasia and carcinoma in situ. However, they did not further analyze the prognosis and clinical characteristics of patients with the up-regulation of Fascin.

Furthermore, we discovered the relationship between high levels of FSCN1 and clinical pathological characteristics of TSCC patients. Our study demonstrated that high FSCN1 expression was correlated with local metastasis and late clinical stage. In addition, the TSCC patients with high FSCN1 expression were more likely to relapse. Consistent with our findings, Chen et al. and Alam et al. reported that Fascin protein expression was correlated with positive lymph node metastasis and clinical staging in oral squamous cell carcinoma^19,24^. Additionally, Lee et al. found that Fascin protein expression is also related to tumor recurrence^21^.

The above results suggested that tumors with high FSCN1 levels might be more aggressive and may forecast poor prognosis in TSCC patients. Our Kaplan-Meier survival analysis confirmed this hypothesis that TSCC patients with high FSCN1 expression had a higher risk of recurrence and shorter DFS. Though our OS was no statistical significance, its *p* value was close to 0.05. If we expanded the sample size, the results might be more convincing. The TCGA statistic supported our hypothesis in head and neck squamous carcinoma. Cox hazard ratio regression analysis further confirmed that FSCN1 expression, together with clinical stage, is an independent risk factor in TSCC patients.

The results in human beings were consistent with the results in vitro and in vivo. Over-expression of FSCN1 was related to aggressive characteristic and poor prognoses in TSCC patients. Therefore, the examination of FSCN1 expression by immunohistochemistry may be a reliable tool for the prediction of risk of recurrence or progression, and it may help optimize individual therapy for TSCC patients.

Our findings prove that FSCN1 is a potential therapeutic target in TSCC. Target-specific anti-Fascin agents are a potential therapy for treatment in TSCC, which may open new avenues for the development of antineoplastic drugs. Some recent studies have found that some microRNAs (miRNAs/miRs) could inhibit proliferation, migration or invasion via targeting FSCN1 in different cancers, such as miR-200b and microRNA-133b in non-small cell lung cancer^25,26^, microRNA-663 in colorectal cancer^27^, miR-539 in hepatocellular carcinoma^28^ and miR-145-5p in laryngeal squamous cell carcinoma^29^. Han et al. reported the development of Fascin-specific small molecules (NP-G2-011 and NP-G2-044) that inhibit the interaction between Fascin and actin. These inhibitors could block tumor cell migration and tumor metastasis. Mechanistically, these inhibitors likely occupy one of the actin-binding sites, reduce the binding of actin filaments, and thus lead to the inhibition of the bundling activity of Fascin^30^. However, the role of these Fascin-specific small molecules in TSCC needs to be further verified. Rodrigues et al. explored the Correlation between Fascin and miR-138 and miR-145 expression in oral squamous cell carcinoma and finally found that forced expression of miR-138 in oral squamous cell carcinoma cells significantly decreased the expression of Fascin^6^. Nevertheless, there are still no FSCN1 inhibitors available in clinical trials or clinical treatment. So there is still a lot of work to find targeted drugs for accurate treatment of TSCC, including the exploration of more Fascin-specific small molecules and further validation in clinic.

Many scholars have studied the molecular mechanism of FSCN1 in many different cancers. Previously, researchers had analyzed FSCN1 expression in cancers, such as esophageal, gastric, and oral carcinoma^7,10,21^. These studies found that FSCN1 was identified at the invasive borders of the tumor cell nests in most of the cancers mentioned above. This indicated that the Fascin protein originally induced morphological transformation in cell membrane protrusions, reduced intercellular adhesion, and finally improved the motility of tumor cells to promote micro-invasion, early and advanced invasion, and metastasis. Huang et al. argued that Fascin played an important role in communicating with the extracellular microenvironment and in fundamental cell functions, such as cell adhesion, spreading, and migration in the three-dimensional environment^31^. Monther et al. first showed that Fascin down-regulated the expression and nuclear translocation of a key metastasis suppressor protein known as breast cancer metastasis suppressor-1 (BRMS1). In addition, Fascin up-regulates NF-kappa B activity and up-regulates other proteins that are known to be critical for the execution of metastasis such as urokinase-type plasminogen activator (uPA) and the matrix metalloproteases (MMP)-2 and MMP-9^32^. Moreover, they found that Fascin is Critical for the maintenance of breast cancer stem cell pool predominantly via the activation of the notch self-renewal pathway^33^. In addition, Lin et al.’s data demonstrated that monoubiquitination decreased the Fascin bundling EC50, delayed the initiation of bundle assembly and accelerated the disassembly of existing bundles^34^. Alam et al. found that Fascin promotes tumor progression and activates AKT and MAPK pathways in OSCC-derived cells^19^. Rodriges et al. found that Fascin may have a major role in OSCC migration and invasiveness process through in vivo and in vitro experiment and forced expression of miR-138 in OSCC cells decreased the expression of Fascin^6^. But the exact mechanism of FSCN1 in the behavior of TSCC requires further study.

## Conclusion

In summary, our study revealed that FSCN1 is over-expressed in TSCC, which is related to poor clinical outcomes. Knockdown of FSCN1 correlated with lower progression and less trans-migration in vitro and decreased tumor growth in vivo. Our results suggest that FSCN1 is an effective marker of poor prognosis and a potential therapeutic target in TSCC. The mechanisms of FSCN1 up-regulation in TSCC and its role in carcinogenesis require further exploration.

## Supplementary information

Clinical characteristic and FSCN1 express of 106 patient samples of TSCC
