# Supplementary material for: FSCN1 is an effective marker of poor prognosis and a potential therapeutic target in human tongue squamous cell carcinoma
Source: Cell Death Dis. 2019 May 1;10(5):356. doi: 10.1038/s41419-019-1574-5 (PMC6494834; doi:10.1038/s41419-019-1574-5)
Supplement: Supplementary file 1 — Clinical characteristic and FSCN1 express of 106 patient samples of TSCC [file 41419_2019_1574_MOESM1_ESM.docx]

Supplemental Table.1 Clinical characteristic and FSCN1 express of 106 patient samples of TSCC

| **Characteristics** |  |  |  |  | **Number of cases (%)** | | | |  |
| --- | --- | --- | --- | --- | --- | --- | --- | --- | --- |
| **Age (years)** |  |  |  |  |  |  |  |  |  |
| **≤45** |  |  |  |  | 27(25.5) | | |  |  |
| **>45** |  |  |  |  | 79(74.5) | | |  |  |
| **Gender** |  |  |  |  |  |  |  |  |  |
| **Male** |  |  |  |  | 62(58.5) | | |  |  |
| **Female** |  |  |  |  | 44(41.5) | | |  |  |
| **Differentiation stage** | | |  |  |  |  |  |  |  |
| **Well** |  |  |  |  | 72(67.9) | | |  |  |
| **Moderate** |  |  |  |  | 28(26.4) | | |  |  |
| **Poor** |  |  |  |  | 6(5.7) | | |  |  |
| **T classification** |  |  |  |  |  |  |  |  |  |
| **T1** |  |  |  |  | 42(39.6) | | |  |  |
| **T2** |  |  |  |  | 41(38.7) | | |  |  |
| **T3** |  |  |  |  | 22(20.8) | | |  |  |
| **T4** |  |  |  |  | 1(0.9) | | |  |  |
| **N classification** | |  |  |  |  |  |  |  |  |
| **N0** |  |  |  |  | 80(75.5) | | |  |  |
| **N1** |  |  |  |  | 12(11.3) | | |  |  |
| **N2** |  |  |  |  | 14(13.2) | | |  |  |
| **N3** |  |  |  |  | 0 | | |  |  |
| **M classification** | |  |  |  |  |  |  |  |  |
| **M0** |  |  |  |  | 105(99.1) | | |  |  |
| **M1** |  |  |  |  | 1(0.9) | | |  |  |
| **Clinical stage** |  |  |  |  |  |  |  |  |  |
| **I** |  |  |  |  | 37(34.9) | | | |  |
| **II** |  |  |  |  | 31(29.2) | | | |  |
| **III** |  |  |  |  | 21(19.8) | | | |  |
| **IV** |  |  |  |  | 17(16.1) | | | |  |
| **Relapse** |  |  |  |  |  | | | |  |
| **Yes** |  |  |  |  | 30(28.3) | | | |  |
| **No** |  |  |  |  | 76(71.7) | | | |  |
| **Death** |  |  |  |  |  | | | |  |
| **Yes** |  |  |  |  | 32(30.2) | | | |  |
| **No** |  |  |  |  | 74(69.8) | | | |  |
| **Expression of FSCN1** | | |  |  |  |  |  |  |  |
| **Low expression** | |  |  |  | 37(34.9) | | | |  |
| **High expression** | |  |  |  | 69(62.2) | | | |  |
